# Editorial: Digital Health in Cardiovascular Medicine

**DOI:** 10.3389/fcvm.2021.810992

**Published:** 2021-12-09

**Authors:** Stefano Omboni, Bela Benczur, Richard J. McManus

**Affiliations:** ^1^Clinical Research Unit, Italian Institute of Telemedicine, Varese, Italy; ^2^Department of Cardiology, Sechenov First Moscow State Medical University, Moscow, Russia; ^3^First Department of Internal Medicine (Cardiology-Nephrology), Balassa Janos County Hospital, Szekszard, Hungary; ^4^Nuffield Department of Primary Care Health Sciences, University of Oxford, Oxford, United Kingdom

**Keywords:** digital health, cardiovascular disease, telemedicine, m-health, wearables

Digital health is defined as the use of information and communications technologies to manage patients and their health and promote their wellness by making communication between caregivers and patients more efficient (1). The broad scope of digital health includes several applications used for storing, retrieving, sharing, and exchanging health-related information for prevention, diagnosis, treatment, monitoring, educational, and administrative purposes. Three major predicted areas of disruption in healthcare models through digital health are telemedicine, mobile health (m-health), and wearable sensors. These great value categories of digital health are the main object of the article collection in the Research Topic.

As highlighted in the review by Omboni, the most popular digital health application for hypertension management is blood pressure telemonitoring, which has been shown to improve blood pressure control, increase treatment adherence, and favor intensification and optimization of antihypertensive medications. Among the available telemedicine solutions, those based on m-health, providing medication tracking and reminding, teleconsultation, and cuffless blood pressure measurement seem to be the most promising tools.

As pointed out in the article by Bard et al., the recent introduction of cuffless blood pressure measurement solutions has the potential to allow a more extensive adoption of blood pressure telemonitoring by enhancing patient autonomy, favoring self-management, and providing physicians with a more complete picture of their patients' blood pressure profile. Although cuffless tools based on applications that enable smartphones to measure blood pressure or rely on wearables are very promising, their routine use is challenged by several potential drawbacks, including a lack of accepted validation schedules, the need to calibrate through conventional means, questionable accuracy, and lack of comparability with office or cuff-based home blood pressure measurement (2).

Smartwatches are also currently very popular for other applications in the cardiological field, where they are used for tracking blood pressure, heart rate, and cardiac arrhythmias. Baka and Simko suggest that wrist-worn devices that can continuously monitor cardiac rhythm and heart rate over the 24 h may be helpful to identify patients with a non-dipping heart rate pattern at night, associated with an increased risk of cardiovascular complications.

A topical application of smartwatches is the screening of atrial fibrillation. However, as discussed by Predel and Steger in their article, the lack of evidence for an improved outcome, the high number of false-positive results, inadequate data protection laws, and the lack of sufficient user education make their use in such screening questionable, unless patients are carefully selected and supervised by a doctor. Current guidelines recommend that when atrial fibrillation is detected by a mobile device or wearable screening tool, a single-lead ECG tracing of at least 30-s duration or 12-lead ECG showing atrial fibrillation evaluated by a physician with expertise in ECG rhythm interpretation must be provided to establish a definitive diagnosis (3). In the lack of this confirmatory ECG recording, the suspected diagnosis of atrial fibrillation cannot be accepted, and oral anticoagulant therapy is not allowed to be initiated.

Difficulty using the technologies and lack of adequate infrastructures are major challenges to effective digital health uptake among patients (4). A possible solution to these critical drawbacks is embedding multimodal sensors in home appliances that enable the collection of information about physical activity, sleep quality, and vital signs during daily life. In their original research paper, including 20 patients and in one case report, Saner et al. indicate that monitoring community-dwelling seniors in their home or apartment with a multimodal ambient and wearable pervasive computing system over more than 1 year is feasible and well-accepted. It helped to capture and promptly manage several episodes of health deterioration, particularly heart failure worsening and heart rhythm disturbances. How scaleable such approaches are in 2021 is questionable, but by 2031 such infrastructure may sit alongside smart thermostats, door bells, or lighting systems, hence lowering the bar for access considerably.

A typical digital health application is represented by remote video consultation between doctor and patient (so-called televisits) (5). Kamel et al. evaluated the impact of such an intervention in a group of 200 acute STEMI patients following primary PCI. During the 3 month study period, patients receiving monthly videoconferencing teleconsultation through a smartphone plus at least a single face-to-face clinic visit showed better adherence to medications (statins, angiotensin-converting enzyme inhibitors, or angiotensin receptor blockers) and healthy lifestyle measures (smoking cessation and cardiac rehabilitation) than patients who received only clinic visits over the same period. Patients rated virtual visits with a high level of satisfaction.

In conclusion, the articles presented in this Research Topic confirm that digital health, particularly telemedicine and m-health, is transforming the management of cardiovascular conditions such as hypertension, atrial fibrillation, coronary artery disease, and heart failure by healthcare professionals and patients alike. The current COVID-19 health emergency has accelerated this digitalization of healthcare systems, dramatically reducing the occurrence of face-to-face contact between patients and their healthcare managers (6). Whether the benefits in the management of cardiovascular patients brought about by digital health can be sustained over the long haul and beyond the current pandemic need to be elucidated in future studies.

## Author Contributions

SO prepared the editorial. RJM and BB critically revised its content. All authors meet the ICMJE criteria for authorship for this manuscript, take responsibility for the integrity of the work as a whole, and have given final approval to the version to be published.

## Conflict of Interest

SO was a scientific consultant of Biotechmed Ltd., a provider of telemedicine services. RJM was working with Omron to develop and evaluate the Hypertension Plus Telemonitoring system. The remaining author declares that the research was conducted in the absence of any commercial or financial relationships that could be construed as a potential conflict of interest.

## Publisher's Note

All claims expressed in this article are solely those of the authors and do not necessarily represent those of their affiliated organizations, or those of the publisher, the editors and the reviewers. Any product that may be evaluated in this article, or claim that may be made by its manufacturer, is not guaranteed or endorsed by the publisher.

## References

[1] Omboni S. Connected health: in the right place at the right time. Conn Health. (2021) 1:1–6. 10.20517/ch.2021.01

[2] Stergiou GS Palatini P Parati G O'Brien E Januszewicz A Lurbe E. 2021 European Society of Hypertension practice guidelines for office and out-of-office blood pressure measurement. J Hypertens. (2021) 39:1293–302. 10.1097/HJH.0000000000002843 33710173

[3] Hindricks G Potpara T Dagres N Arbelo E Bax JJ Blomstrom-Lundqvist C. 2020 ESC Guidelines for the diagnosis and management of atrial fibrillation developed in collaboration with the European Association for Cardio-Thoracic Surgery (EACTS): The Task Force for the diagnosis and management of atrial fibrillation of the European Society of Cardiology (ESC) Developed with the special contribution of the European Heart Rhythm Association (EHRA) of the ESC. Eur Heart J. (2021) 42:373–498. 10.1093/eurheartj/ehaa612 32860505

[4] Whitelaw S Pellegrini DM Mamas MA Cowie M Van Spall HGC. Barriers and facilitators of the uptake of digital health technology in cardiovascular care: a systematic scoping review. Eur Heart J Digit Health. (2021) 2:62–74. 10.1093/ehjdh/ztab005 34048508 PMC8139413

[5] Li CZ Borycki EM Kushniruk AW. Connecting the world of healthcare virtually: a scoping review on virtual care delivery. Healthcare. (2021) 9:1325. 10.3390/healthcare9101325 34683005 PMC8544348

[6] Omboni S Ballatore T Rizzi F Tomassini F Panzeri E Campolo L. Telehealth at scale can improve chronic disease management in the community during a pandemic: An experience at the time of COVID-19. PLoS ONE. (2021) 16:e0258015. 10.1371/journal.pone.0258015 34587198 PMC8480747

